# HIV rapid testing in community and outreach sites: results of a nationwide demonstration project in Italy

**DOI:** 10.1186/s12889-018-5680-6

**Published:** 2018-06-18

**Authors:** Paola Scognamiglio, Giacomina Chiaradia, Marta Giovanetti, Emidio Albertini, Antonella Camposeragna, Massimo Farinella, Daniela Lorenzetti, Massimo Oldrini, Laura Rancilio, Anna Caraglia, Francesco Paolo Maraglino, Giuseppe Ippolito, Enrico Girardi

**Affiliations:** 1 Clinical Epidemiology Unit - Department of Epidemiology and Preclinical Research, National Institute for Infectious Diseases “L. Spallanzani”, IRCCS, Via Portuense 292, 00149 Rome, Italy; 2 Onphalos LGBTI (Lesbian, Gay, Bisexual, Transgender/Transsexual and Intersexed), Perugia, Italy; 3 CNCA - National Coordination of Care Communities, Rome, Italy; 4 “Mario Mieli” Homosexual Cultural Circle, Rome, Italy; 5 ANLAIDS Onlus - Italian National Association for the fight against Aids, Rome, Italy; 6 Lila Onlus - Italian League for the Fight against AIDS, Milan, Italy; 7 Caritas Ambrosiana, AIDS, Addictions and Mental Health Area, Milan, Italy; 8 Directorate-general for Health Prevention, Infectious Diseases Office, Ministry of Health, Rome, Italy

**Keywords:** HIV rapid test, Community-based services, Counseling and testing, Acceptability

## Abstract

**Background:**

Globally the access to HIV testing has greatly increased over the past 30 years. Nonetheless, a high proportion of people living with HIV remains undiagnosed, even in resource rich countries. To increase the proportion of people aware of their HIV serostatus and their access to medical care, several strategies have been proposed including HIV rapid test programs offered outside health facilities. The aim of this project was to evaluate the feasibility and efficacy of the HIV rapid testing offered in community and outreach settings in Italy.

**Methods:**

We conducted a national demonstration project on HIV rapid tests offered in community and outreach settings, including nongovernmental organization (NGO) facilities, primary care services for migrants and low-threshold services or mobile units for drug users (DU services). HIV rapid test on oral fluid (OraQuick®; Orasure Technologies) was anonymously offered to eligible people who presented themselves at the selected sites. Those with reactive results were referred to a specialized outpatient unit for confirmatory testing and medical care.

**Results:**

Over a period of six months a total of 2949 tests were performed and 45.2% of individuals tested had not been previously tested. Overall 0.9% (27/2949) of tested people had a preliminary positive test. In NGO facilities the positivity rate was 1%. All subjects who performed their confirmatory test were confirmed as positive. In services for migrants the positivity rate was 0.5 and 80% were referred to care (with 1 false positive test). In DU services we observed the highest positivity rate (1.4%) but the lowest linkage to care (67%), with 1 false positive test.

**Conclusion:**

Our project showed that the offering of an HIV rapid testing program in community and outreach settings in Italy is feasible and that it may reach people who have never been tested before, while having a significant yield in terms of new HIV diagnoses as well.

## Background

Interventions aimed at promoting access for people living with HIV (PLWH) to diagnosis, treatment and viral suppression have emerged in the last decade as central elements in the strategies for controlling, and eventually ending, the AIDS epidemic [1, 2]. In this context, the Joint UN Program on HIV/AIDS (UNAIDS) set the HIV 90–90-90 target, whereby 90% of PLWH should be diagnosed, 90% of those diagnosed should receive antiretroviral treatment and 90% of those treated should achieve viral suppression globally by 2020 [3].

Globally the access to HIV testing has greatly increased over the past 30 years, nonetheless, it was estimated that in 2014 more than 40% of PLWH remained undiagnosed, especially in resource limited countries [4] and that more efforts are needed to address this issue, even in resource rich countries. An analysis based on CD4 back-calculation and on HIV/AIDS surveillance data showed that in the United States 16.4% of PLWHIV were undiagnosed in 2013 [5]. By using a similar approach, it was estimated that in the same year 16% of all PLWHIV had had undiagnosed infection in 11 European Union (EU) countries [6]. Reducing the size of HIV undiagnosed population will be critical in the progression towards the control of the epidemic since those people who are HIV infected but undiagnosed contribute disproportionately to HIV transmission [7, 8]. A recent study has shown that in Netherlands 71% of transmissions among men having sex with men in last few years were from undiagnosed men [9].

The development of HIV rapid testing technology has created new opportunities for promoting HIV testing interventions outside the formal health facilities. These interventions have been designed to target specific groups of the population who are most at risk of infection. They have been defined as community based testing, and may allow the involvement of community representatives [10, 11]. Implementation of these testing services has been recommended by such health agencies, as the World Health Organization (WHO) and the European Center for Disease Prevention and Control (ECDC), on the basis of evidence supporting either their role in addressing important barriers to testing encountered within the formal health care system or in promoting early diagnosis among those most at risk [12, 13].

However, a wide range of variation exists in the implementation of Community Based Voluntary Counselling and Testing (CBVCT) services. For example in a survey conducted in the EU in 2011–2012, 20 of the 25 National Focal Points (NFPs) participating in the survey reported the availability of the CBVCTs in their Countries, and the estimated number varies from 50/100 CBVCT services reported by UK NFP to 1 service reported by Slovenian and Portuguese NFP. Availability of CBVCT in Italy was not reported in that survey [10]. In Italy, HIV testing opportunities are frequently limited to the hospital setting and few initiatives have been proposed to validate the use of rapid diagnostic tests in other contexts [14].

We conducted a national demonstration project on the HIV rapid test offered outside the formal health facilities in collaboration with six non-governmental organizations (NGOs) in order to assess feasibility and potential yields of CBVCT implementation in different settings. This article reports the main finding of this project.

## Methods

The project was based on a partnership between the National Institute for Infectious Diseases and six Italian NGOs working in the field of HIV/AIDS. A total of 23 sites for testing were identified in nine different Regions, evenly distributed in the whole National territory; these sites included NGOs facilities (*n* = 10) primary care services for migrants (*n* = 8) and low-threshold services or mobile units for drug users (DU services) which provide drug treatment and harm reduction (*n* = 5), all run by an NGO. All the selected services had proven previous experience in HIV counseling and were able to guarantee respect for testing privacy and confidentiality of all testing procedures. For each site, a team was identified that included at minimum a lay counselor and a certified healthcare worker who, according to the Italian Law, are permitted to perform diagnostic testing. Training sessions were organized for team members, and each site established links with local infectious disease clinics to ensure follow up visits for those people testing positive for test confirmation and initiation of care. During a period of 3 months, each NGO scheduled a calendar of dates for HIV test offers. In particular, in NGO facilities testing was performed (by appointment) in designated days and this was advertised on the internet and in locations such as bars or during special events. In migrants and DU services HIV testing was offered to all eligible people attending the services during the study period on the days in which the project team was present. People were eligible for HIV testing if they reported being HIV-negative or did not know their HIV status, if they were more than 18 years old (the age requirement for consent in Italy), or if they were able to provide informed consent.

Those people who refused the study were asked to fill in a short questionnaire investigating their reasons for rejection.

Trained counsellors provided HIV pre-test informative counselling to all those who agreed to be tested. Pre-test counselling included: basic information on HIV/AIDS; description of testing procedures (need of informed consent and administration of risk assessment questionnaire, anonymity); characteristics of the test performed and reliability of test results; need of confirmatory testing and clinical follow-up in case of reactive results; clinical services available for confirmatory testing and clinical follow-up.

In this project, we used an HIV rapid test performed on oral fluid (OraQuick Advance® Rapid HIV-1/2 Antibody Test; OraSure Technologies Inc.) which provides results in 20 min. While waiting for test result, an anonymous questionnaire was administered. The questionnaire collected socio-demographic data and information on HIV testing history, recent sexually transmitted infections (STI) and sexual and drug use behaviour. Testing procedures were organized in order to respect confidentiality and all services arranged a specific room for counselling and all testing procedures (provision for counselling, test performance, administration of a risk assessment questionnaire, and communication of test results). For mobile units, all testing procedure were run in close units (e.g. in a camper) and users were tested one by one.

The healthcare operator communicated the test result and provided post-test counselling. People with reactive tests were referred to the clinical centres linked to the project for confirmatory testing and initiation of care.

To estimate the effectiveness of the intervention we used the following process and outcome measures: coverage: number of people approached/total of service users; testing rate: number of tested people/total number of approached people; positivity rate: people with reactive test/total number of tested people; linkage to care: people attending the referral clinical center linked to the project for confirmatory testing and first visit /people with reactive testing. We also analyzed the proportion of first-time testers, i.e. people reporting no previous HIV tests, and prevalence of at risk behaviors among those tested. To compare socio-demographic characteristics and risk behaviors of first time testers with repeat testers and people with reactive test vs people with negative tests we performed a t test and a chi-square test, as appropriate, excluding those with missing data from the analysis.

Gathered data were anonymously registered through the use of the identification code in a Access database and analyzed using SPSS software (version 18.0).

The project, and the relative data collection, was approved by the Ethical committee at the National Institute for Infectious Diseases.

## Results

The project was carried out between February and July 2013 and a total of 2949 tests were performed in 427 days of testing. In NGO facilities, the test was offered for a median of 20 days, in migrant services for a median of 22 days and in DU services for a median of 8 days.

### HIV testing process

**Fig. 1:**
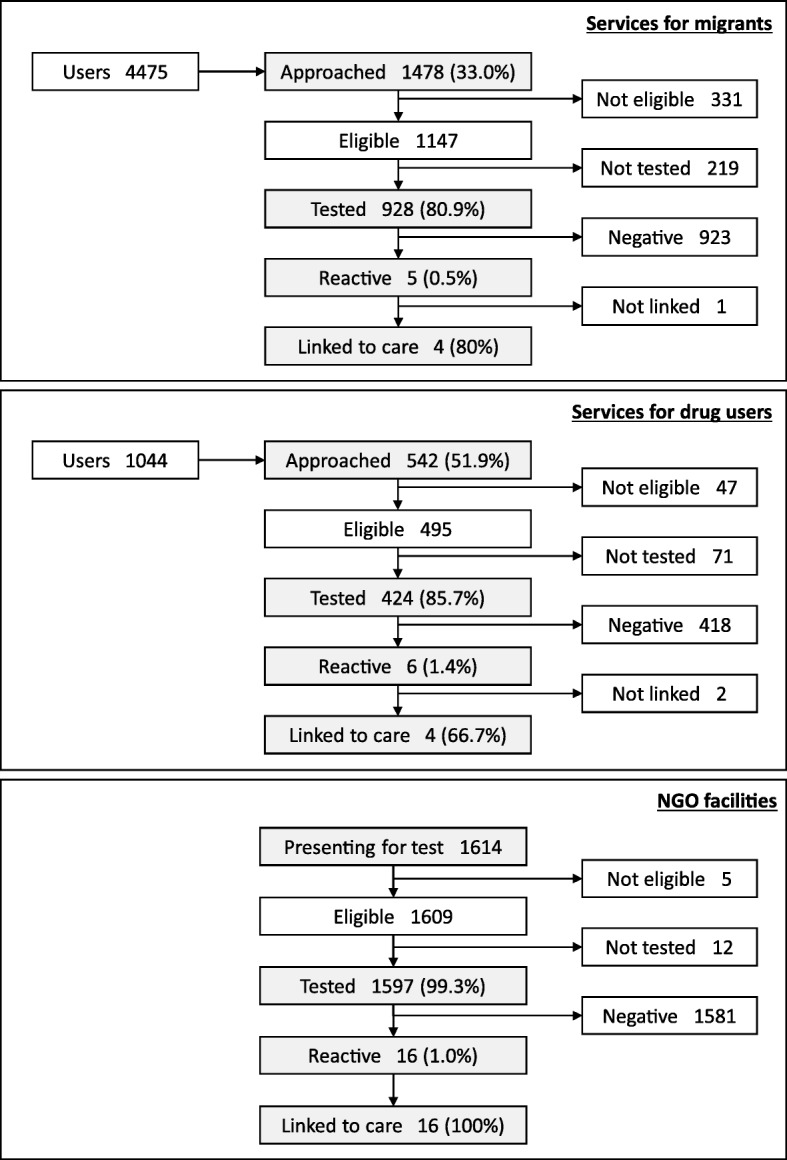
Flow of people in the project

The flow of people in the project is shown in fig. 1. During the study period, 4475 people attended the services for migrants and 1044 the DU services. Of these 1478 (33%) and 542 (52%) respectively for services for migrants and DU services were actually approached for the offering of the test, for an overall coverage of the program in these sites of 37%. Of those approached and found eligible in these sites 928/1147 (81%) and 424/495 (86%) respectively accepted being tested with an overall testing rate of 1352/1642 (82%). Of the 290 persons who refused testing in these sites, 54 (19%) agreed to fill in a brief questionnaire. The most frequently reported reasons for refusing testing were to have already been recently tested (35%) and not to have had risk behaviors (20%).

In addition, 1597 people were tested in NGO facilities, for a total of 2949 people tested in this project.

### Sociodemographic and behavioural characteristics of the people tested

Socio-demographic characteristics and information on the sexual behavior and drug use of those people tested, overall and stratified by setting type are shown in Tables 1 and 2.

**Table 1:** Socio-demographic characteristics of tested population (overall and stratified by type of setting)

	Tested population	NGO facilities	Services for migrants	DU services
	*N* = 2949 (%)	*N* = 1597 (%)	*N* = 928 (%)	*N* = 424 (%)
Median age (IQR)	33 years (26–43)	31 years (25–41)	37 years (29–47)	35 years (28–44)
Gender				
Men	1934 (65.6)	1123 (70.3)	531 (57.2)	280 (66. 0)
Women	973 (33)	445 (27.9)	391 (42.1)	137 (32.3)
Transgender	32 (1.1)	23 (1.5)	4 (0.4)	5 (1.2)
Unknown	10 (0.3)	6 (0.4)	2 (0.2)	2 (0.5)
Origin				
Italian	1782 (60.4)	1432 (89.7)	86 (9.3)	264 (62.3)
Foreigners	1167 (39.6)	165 (10.3)	842 (90.7)	160 (37.7)
Education (yy)				
≤ 8	684 (23.2)	167 (10.5)	340 (36.6)	177 (41.7)
> 8	2249 (76.3)	1421 (88.9)	583 (62.8)	245 (57.8)
Unknown	16 (0.5)	9 (0.6)	5 (0.6)	2 (0.5)
Occupation				
Employed	1478 (50.1)	939 (58.8)	413 (44.5)	126 (29.7)
Occasional employed	485 (16.4)	426 (26.7)	37 (4)	22 (5.2)
Unemployed	968 (32.8)	223 (14)	472 (50.9)	273 (64.4)
Unknown	18 (0.6)	9 (0.6)	6 (0.6)	3 (0.7)
Marital status				
Never married	1895 (64.3)	1283 (80.3)	374 (40.3)	238 (56.1)
Married/Cohabiting	694 (23.5)	206 (12.9)	378 (40.7)	110 (25.9)
Married before	305 (10.3)	78 (4.9)	156 (16.8)	71 (16.7)
Unknown	55 (1.9)	30 (1.9)	20 (2.2)	5 (1.2)
Previous HIV test				
No	1334 (45.2)	565 (35.4)	613 (66.1)	156 (36.8)
Yes	1545 (52.4)	1016 (63.3)	283 (30.5)	246 (58.0)
Unknown	70 (2.4)	16 (1.0)	32 (3.4)	22 (5.2)
Previous STI^a^				
No	2406 (81.6)	1289 (80.7)	777 (83.7)	431 (80.4)
Yes	392 (13.3)	257 (16.1)	90 (9.7)	45 (10.6)
Unknown	151 (5.1)	52 (3.3)	61 (6.6)	38 (9.0)

*IQR* interquartile range, *NGO* nongovernmental organization, *DU* drug users, *STI* sexually transmitted infection

^a^in previous 12 months

**Table 2:** Sexual and drug using behaviors of tested population (overall and stratified by type of setting)

	Tested population	NGOfacilities	Services for migrants	DU services
	*N =* 2949 (%)	*N =* 1597 (%)	*N =* 928 (%)	*N =* 424 (%)
Sexual behaviors				
Heterosex	1519 (51.5)	626 (39.2)	608 (65.5)	285(67.2)
Male SGS	794 (26.9)	766 (48)	14 (1.5)	14 (3.3)
Condom in the last sexual intercourse^a^				
No	1826 (61.9)	1022 (64.0)	534 (57.5)	270 (63.7)
Paying for sex^a^				
Yes	507 (17.2)	249 (15.6)	123 (13.2)	135 (31.8)
Prostitution (sex for money)^a^				
Yes	242 (8.2)	112 (7.0)	49 (5.3)	81 (19.1)
Sex under influence of drugs or alcohol^a^				
Yes	916 (31.1)	552 (34.6)	121 (13.0)	243 (57.2)
Drug use^a^				
Intravenus drug use	135 (4.6)	19 (1.2)	11 (1.2)	105 (24.8)
Not intravenus drug use	675 (22.8)	448 (28.1)	63 (6.8)	164 (38.7)
Drug use way not known	132 (4.5)	97 (6.1)	19 (2.0)	16 (3.8)

*NGO* nongovernmental organization, *DU* drug users, *SGS* Same gender sex

^a^in previous 12 months

Subjects tested in the NGO facilities had a median age of 31 years (interquartile range – IQR: 25–41), were largely male (70%) and Italian (90%). A total of 48% of the people tested in this setting were male who reported same gender sex, 64% reported not using condom during their last sexual intercourse and 1.2% declared intravenous drug use in the previous 12 months. People tested in healthcare services for migrants mainly came from Eastern Europe (35%), North Africa (18%) and Sub-Saharian Africa (18%). They had a median age of 37 years (IQR 29–47) and 57.2% of them was male. A total of 65.5% reported being heterosexual and 57.5% reported not using condom during their last sexual intercourse. 1.2% of those tested declared intravenous drug use in the previous 12 months.

Persons tested in DU services were mainly males (66%), had a median age of 35 years (IRQ 28–44) and the 37.7% were foreign born, mainly coming from Eastern Europe and North Africa. A total of 67.2% were self-declared heterosexual, 63.7% reported not using condom during their last sexual intercourse and 67.3% reported the use of drugs in the previous 12 months (24.8% intravenous consumers).

Among people tested, 1334 (45.2%) reported they had not been previously tested. The proportion of first testers was higher in migrant services (66.1%). The characteristics of first testers are described in Table 3. First testers had a median age of 30.5 years and were largely male (56.8%), of foreign origin (52.8%). Regarding risk factors for HIV, 10% of first testers referred a previous STI, 18.6% identified themselves as homosexual, the majority (61.8%) declared not using condom during their last sexual intercourse, 5.7% prostituted themselves in the previous 12 months and 29.2% reported using drugs in the previous year (1.2% intravenous). Comparing characteristics and risk behaviors of first testers with repeat testers, first testers were significantly younger (*p* < 0.001). Moreover, being a first tester was significantly associated with female gender (58.5% vs 39.2% for males, *p* <  0.001), foreign origin (60.3% vs 35.4% for those born in Italy, *p* <  0.001), low education level (52.9% vs 43.0% for those with > 8 years of education, *p* <  0.001), being unemployed/not in labor force (50.9% vs 39.6% for those employed, *p* <  0.001), being married (54.0% vs 41.9% for unmarried *p* <  0.001), reporting no previous STIs (45.6% vs 34.9%, *p* <  0.001), not-paying for sex (45.9% vs 37.5%, *p* <  0.001), not being sex worker (45.9% vs 31.4% for sex workers, *p* <  0.001), not having sex under influence of drugs or alcohol (46.6% vs 41.4% for those reporting this behavior, *p* = 0.008) and no drug use, both intravenous and non-intravenous (47.9% vs 38.4% for those reporting drug use, *p* <  0.001).

**Table 3:** Characteristics and sexual/drug using behaviors of first-time testers

First testers	*N* = 1334 (%)
Median age (IQR)	30.5 (24–41) years
Gender	
Men	758 (56.8)
Women	569 (42.7)
Transgender	1 (0.1)
Unknown	6 (0.4)
Setting	
NGOs facilities	565 (42.4)
Services for migrants	613 (46.0)
DU services	156 (11.7)
Origin	
Italian	630 (47.2)
Foreigners	704 (52.8)
Education (YY)	
≤ 8	362 (27.1)
> 8	967 (72.5)
Unknown	5 (0.4)
Marital Status	
Never married	794 (59.5)
Married/Cohabiting	375 (28.1)
Married before	136 (10.2)
Unknown	29 (2.2)
Previous STI^a^	
	137 (10.3)
Sexual behaviors	
Heterosex	791 (59.3)
Male SGS	184 (13.8)
Condom in the last sexual intercourse^a^	
No	824 (61.8)
Paying for sex^a^	
Yes	190 (14.2)
Prostitution^a^	
Yes	76 (5.7)
Sex under influence of drugs or alcohol^a^	
Yes	379 (28.4)
Drug use^a^	
Intravenus drug use	16 (1.2)
Non intravenous drug use	297 (22.2)
Drug use way not known	49 (3.7)
Preliminary positive test	8 (0.6%)

*IQR* interquartile range, *NGO* nongovernmental organization, *DU* drug users, *STI* sexually transmitted infection, *SGS* same gender sex

^a^in previous 12 months

### Project yield

Overall, the positivity rate was 0.9% (27/2949). In NGO facilities the positivity rate was 1% (16/1597). All subjects with preliminary positive testing in this setting were linked to referral specialized centers for confirmatory testing and first visit (linkage to care = 100%). All reactive tests were confirmed as positive. In services for migrants the positivity rate was 0.5% (5/928) and 4/5 of those with reactive test attended centers linked to the project for confirmatory testing and first visit (linkage to care = 80%), with 1 false positive test. In DU services we observed the highest positivity rate (1.4%) but the lowest linkage to care (67%), with 1 false positive test. No further information was available for those people with preliminary positive testing who did not attended the centers linked to the project for confirmatory testing.

Among the 27 people with new HIV diagnoses, 85% were male and 33% were of foreign origin. 13 (48%) identified themselves as homosexual and 7 (26%) reported using injected drugs in the previous year. 30% of newly diagnosed subjects (confirmed as positive) had never been tested before. We found no significant association between having a reactive test and socio-demographic characteristics or reported risk.

## Discussion

Implementation of rapid testing in non-clinical settings as part of community-based interventions is a complementary approach to cope with barriers to HIV testing and to facilitate timely identification of people living with undiagnosed HIV infection [15, 16].

In this project, in Italy over a six-month period, 2949 people were tested for HIV in community and outreach sites and most of them were from key population and/or reported substantial risk behavior types. In fact, 26.9% of the people tested were male same gender sex, 4.6% reported intravenous drug use and 61.9% reported not having used a condom in their last sexual intercourse. In addition, 45.2% reported that they had never been tested for HIV. Overall, almost 1% of those tested were positive. Taken together, and consistently with existing literature, these results suggest the feasibility of this intervention and its potential usefulness in promoting HIV testing among persons at risk.

In some settings however only a part of the target population could be reached. In services for migrants only 33% of service users were approached by NGO personal to offer the test and this proportion was 52% in services for DU. The most likely reasons for the low coverage of the program in these settings were logistical problems and understaffing. Nonetheless, once approached, persons in these settings showed a positive acceptance of the test (81%). These results in the range reported in several studies conducted on community-based HTC approaches among key populations in the USA [17].

Testing of persons who had never tested for HIV was an important aspect of this project as well as in similar projects conducted in the USA [18] and in the European Union [19, 20]. A substantial proportion of these people reported risk behaviors such as lack of condom use in their last sexual intercourse (61.8%) or intravenous drug use (1.2%) and, importantly, 0.6% of first testers were reactive (0.5% confirmed as positive).

As concerned the yield of the intervention in terms of those people tested found to be HIV positive and linked to care, the overall positivity rate resulted 0.9%. This is comparable to results observed in similar projects [18, 20, 21]. Among people tested, the proportion of those who were reactive varied substantially among different setting type. Not unexpectedly, the highest portion of reactive persons was found in DU services (1.4%). In NGO facilities the overall proportion of reactive persons was 1 and 48% of those tested were males who reported having same gender sex. The lowest proportion was found in services for migrants (0.5%). These services were chosen primarily in order to offer testing to persons migrating from countries with generalized epidemics. However, these persons made up only 11% of the 928 tested people in these services.

Linkage to HIV prevention and care services is the final goal of HIV testing activities. Overall considered, the “linkage to care” (defined as attendance at referred centers for confirmatory testing and clinical evaluation) resulted high (89%) but, stratifying by settings, the proportion of linkage to care has not been uniform, with a particular difficulty in engaging care to intravenous drug users. In our project, all people found reactive in NGO facilities were linked to specialized services as well as 80% of those with reactive test in services for migrants. This proportion was below 70% among those DU services. No further information on linkage to care was available for persons with reactive tests who did not attend the clinical centers linked to the project, and we cannot rule out that these individuals actually entered clinical care in other clinical centers which were not involved in the project. In any case, our results on linkage to care of DUs are comparable to results from similar European studies [22, 23]. This data maybe partially due to the difficulty in establishing family networks and social support. This suggests that intervention aimed at favoring linkage to care and, above all, access to antiretroviral therapy for infected people is urgently needed in order to grant a suitable approach to HIV care.

Rapid testing, in addition to having logistic advantages, appears to be acceptable. Preliminary studies have demonstrated high acceptance rates for rapid tests both for users and healthcare professionals because they are not invasive, rapid to perform and easy to use and interpret for non-specialized health operators [23–25]. In this project, we chose to use testing on oral fluids to simplify testing logistics and procedures and to obviate the need to apply specific precautions for blood-borne infections.

Recently the risk of false negative test has been emphasized and this could be due to the lack of sensitivity of the test to detect relatively early stages of HIV infection [26–28]. For this reason, it is very important to inform operators on the possibility of false negatives in those reporting recent exposure, especially when the tests were performed in settings where a significant prevalence of recently acquired infections could be anticipated. In such situations, it is crucial to adequately emphasize the need to repeat HIV testing (preferably with p24 antigen-based ELISA assays) to ascertain infections missed by the initial oral test screening [29].

We do not have a measure of sensitivity of the test in this project. However, diagnostic accuracy studies have shown that the test has a sensitivity of 98.03% and a specificity of 99.7% [29].

Out of 24 reactive tests for which there was a confirmatory test, two eventually proved to be false positive. This finding highlights the importance of any reactive test having to be confirmed by more specific testing and the relevance of providing information on the need for further testing during pre-test counselling. It may also be worth noting that both false positive tests occurred in individuals who had not reported high risk behaviors (one was an heterosexual man, never tested before, who had reported unprotected sexual intercourses with his steady partner; the second reported non-injecting drug use).

### Limitations

One of the limits of this project was due to its lack of information on lifetime risk behaviors of the people tested because the information gathered on risk behaviors were mainly designed to assist in counseling and therefore limited to a more recent period (the previous 12 months). Another limitation of the project was due to the difficulty of tracking access to care for those testing positive. In fact, links were established with local infectious disease clinics for confirmation testing and the initiation of care for people testing preliminary positive, but no information on linkage to care were available for those with positive rapid testing who could have sought care in other clinical centers which were not involved in the project. Moreover, as the test was anonymous, we could not ascertain HIV care initiation for individuals with reactive tests, who were not linked to clinical centers participating in the project. Finally, very limited information was available on those people who refused to be tested, as only 19% of them accepted to fill in the brief questionnaire on socio-demographic characteristics and reasons for refusing the test.

## Conclusion

Our project is one of the first attempts to measure the feasibility and the acceptance of the HIV rapid testing offered in outreach and community settings in Italy. The results are quite encouraging, showing a good feasibility (2949 tested persons over a six months period) and a high intervention acceptance (82%). Our results, together with other studies [17, 20, 30], indicate that community-based interventions of HIV testing could represent a key strategy to favor access to HIV testing. Moreover, since the percentage of people reported as first-time testers was high (45%), our results showed the potential for these interventions to reach people who have never been tested even when reporting risk behaviors. Further studies are needed to evaluate the effectiveness of rapid testing community-based programs in favoring access to tests among key populations and vulnerable groups, also in term of timeliness of diagnosis.

## Abbreviations

CBVCT: Community Based Voluntary Counselling and Testing
DU: Drug user
ECDC: European Center for Disease Prevention and Control
EU: European Union
IQR: Interquartile Range
NFPs: National Focal Points
NGO: Non Governmental Organization
PLWH: People living with HIV
STI: Sexually transmitted infections
UNAIDS: Joint UN Program on HIV/AIDS
WHO: World Health Organization
